# Field recordings of transcranial magnetic stimulation in human brain postmortem models

**DOI:** 10.1097/PR9.0000000000001134

**Published:** 2024-02-16

**Authors:** Charles Quesada, Camille Fauchon, Benjamin Pommier, Florian Bergandi, Roland Peyron, Patrick Mertens, Luis Garcia-Larrea

**Affiliations:** a NeuroPain Team, Centre de Recherche en Neurosciences de Lyon (CRNL), Inserm U1028, CNRS UMR5292, UJM & UCBL, Lyon, France; b Physiotherapy Department, Sciences of Rehabilitation Institute (ISTR), University Claude Bernard Lyon 1, Lyon, France; c University of Medecine Jacques Lisfranc, Anatomy Laboratory, UJM, Saint-Etienne, France; d Neurological Department & CETD, University Hospital, CHU Saint-Etienne, Saint-Etienne, France; e Laboratory of Anatomy, Faculté de Médecine Lyon-est, Université Claude Bernard Lyon 1, Saint-Etienne and Lyon, France; f CETD Neurological Hospital Lyon, Hospices Civils de Lyon, Lyon, France

**Keywords:** Magnetic field, Repetitive transcranial magnetic stimulation, rTMS, Pain, Postmortem model

## Abstract

**Introduction:**

The ability of repetitive transcranial magnetic stimulation (rTMS) to deliver a magnetic field (MF) in deep brain targets is debated and poorly documented.

**Objective:**

To quantify the decay of MF in the human brain.

**Methods:**

Magnetic field was generated by single pulses of TMS delivered at maximum intensity using a flat or angulated coil. Magnetic field was recorded by a 3D-magnetic probe. Decay was measured in the air using both coils and in the head of 10 postmortem human heads with the flat coil being positioned tangential to the scalp. Magnetic field decay was interpreted as a function of distance to the coil for 6 potential brain targets of noninvasive brain stimulation: the primary motor cortex (M1, mean depth: 28.5 mm), dorsolateral prefrontal cortex (DLPFC: 28 mm), secondary somatosensory cortex (S2: 35.5 mm), posterior and anterior insulae (PI: 38.5 mm; AI: 43.5 mm), and midcingulate cortex (MCC: 57.5 mm).

**Results:**

In air, the maximal MF intensities at coil center were 0.88 and 0.77 T for the flat and angulated coils, respectively. The maximal intracranial MF intensity in the cadaver model was 0.34 T, with a ∼50% decay at 15 mm and a ∼75% MF decay at 30 mm. The decay of the MF in air was similar for the flat coil and significantly less attenuated with the angulated coil (a ∼50% decay at 20 mm and a ∼75% MF decay at 45 mm).

**Conclusions:**

Transcranial magnetic stimulation coil MFs decay in brain structures similarly as in air, attenuation with distance being significantly lower with angulated coils. Reaching brain targets deeper than 20 mm such as the insula or Antérior Cingulate Cortex seems feasible only when using angulated coils. The abacus of MF attenuation provided here can be used to adjust modalities of deep brain stimulation with rTMS in future research protocols.

## Abstract 2

Our work shows the need to adapt transcranial magnetic stimulation to the depth of the cortical target, and angulated coil should be preferred for deep cortical targets.

## 1. Introduction

Noninvasive cortical stimulation using magnetic fields (MF) was first described in 1985.^3^ Based on Faraday law, MF induces activity in cortical neurons through a single transcranial magnetic pulse. Technical improvements in the 1990s, notably cooling systems, allowed transcranial magnetic stimulation (TMS) to become repetitive (rTMS) and hence be applied iteratively.

In the past 2 decades, rTMS has been increasingly used to relieve pain^1,17,29^ or to alleviate drug-resistant depression.^5,12^ For pain treatment, the main validated target is the contralateral primary motor cortex (M1), which was also the brain target historically used for invasive (epidural) electrical stimulation.^14,25,37^ The main limitation in these—magnetic or electrical—neuromodulations of M1 is that only half of the patients had an effective pain relief. Therefore, future research on neuromodulation aims to enhance the effects on M1 and to test other brain targets such as the secondary somatosensory (SII) cortex, midcingulate cortex (MCC), posterior insula (PI), anterior insula (AI), and dorsolateral prefrontal (DLPFC) cortex as promising sites for pain relief.^4,8,13,21^

For both invasive and noninvasive procedures, stimulation of the primary motor cortex has the advantage over other cortical areas of having a functional landmark allowing to properly set up the neuromodulation device. The observation of motor responses induced by the stimulation or the recording of motor-evoked potentials (MEPs) demonstrates an effective cortical stimulation of motor neurons, and this motor output is the guarantee of an adequate positioning of the stimulation device. The setting of TMS based on evoked potentials and/or neuronavigated procedure with the individual's anatomical scan is used when targeting “noneloquent” brain areas (ie, with no-motor responses), but the effective dose delivered is poorly estimated. Targeting deep brain areas in noninvasive neuromodulation requires at least an estimation of the MF decay as a function of distance from the coil.

Several studies have described the effects of magnetic pulses over neuronal activation in animal models.^2,22^ Mathematical simulations of MF interactions^30,33,39^ have emphasized the impact of bioelectrical characteristics of cranial tissues and brain layers on magnetic field intensity and distribution. Although the magnetic field distribution has been the subject of considerable research, most of the studies were based on computational models and rarely investigated in vivo preparations.^20–24^ To the best of our knowledge, only one study reported the electric field produced by a single pulse of TMS recorded with intracranial deep electrodes in an epileptic patient, with an estimate of the corresponding magnetic field.^25^ This case report was limited by both the static position of the electrode and the need to stimulate with a very small intensity (7% of the generator output). It has not been replicated so far because of the risks of epileptic seizure or brain damage by heating ferromagnetic components.^18^

To provide more comprehensive data on MF decay during TMS in real situations, we report and compare here recordings of MF in both the air and human postmortem (cadaver) models. Our findings provide a framework to choose the TMS intensity to deliver a proper MF in deep brain targets.

## 2. Methods

### 2.1. Stimulation and recording equipment

In a first experiment, MF was generated through a common flat butterfly coil (Cool-B65 coil, Magventure) and a MAGPRO-X100 generator for records in the air and in postmortem heads. Because another (angulated) coil may be used in clinical practice to reach deep regions such as the motor representation of lower limb, in a second experiment, the MF was generated with a cone-shaped butterfly coil (Cool-DB80, Magventure) that delivered biphasic pulses. Its attenuation was also measured in the air, but not in the cadaver.

Recordings of MF were performed using a dedicated magnetic probe (MAGPROBE-3D Magventure, Farum, Denmark) and a high-frequency oscilloscope (PicoScope 3000 series, St Neots, United Kingdom).

### 2.2. Postmortem model

Postmortem specimens were collected by the laboratories of anatomy at the Faculty of Medicine—Université Claude Bernard Lyon1 (n = 8) and at the Faculty of Medicine Jacques Lisfranc—Université Jean Monnet Saint-Etienne (n = 2). Specimens came from persons who gave their body to science for research purposes. Specimens with history of cranial pathology and/or surgery were excluded, and no obvious cranial abnormality was noted for each head. For each specimen, the demographic characteristics and morphometric parameters were collected. No embalming was used for the brain.

### 2.3. Recording of magnetic field in the air

**Figure 1.**
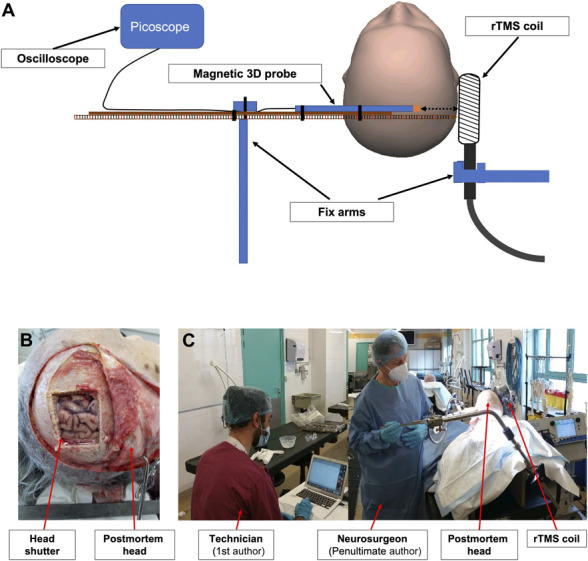
(A) Experimental procedures: schematic representation of the recordings in the cadaver model with the coil placed on the right scalp and the probe introduced inside the postmortem brain from the left to the right side. The same procedure was applied in the air, from a maximal distance of 140 mm to the contact with the coil. (B) Cadaver head with the bone flap and (C) the real experimental conditions when using the cadaver model. rTMS, repetitive transcranial magnetic stimulation.

Each coil (Cool-B65 [flat] and Cool-DB80 [angulated]) was fixed vertically (ie, the handle of the coil was perpendicular to the floor) by a static nonmetallic clip. The recording probe was fixed on a graduated wooden stick that was kept perpendicular to the coil and that was aligned with the center of the coil (Fig. 1A). The probe extremity was adjusted to be in contact with the center of the coil for the first recording position in the air (Pa#1). It was displaced after each stimulus by steps of 5 mm along its supporting stick. The magnetic field was recorded at each position, from full contact with the coil (Pa#1) to a distance of 140 mm (Pa#28). Each TMS stimulus consisted of a single pulse at 100% of the power output of the stimulator. Five consecutive series of measures were conducted with each coil in the air.

### 2.4. Recording of magnetic field in the postmortem model

Cadavers were prepared by an experimented technician from the laboratory of anatomy, and the probe was manipulated by one of the authors (P.M., neurosurgeon). The Cool-B65 rTMS coil was fixed on the right side of the head (Fig. 1B). The center of the coil was adjusted to be in contact with the scalp, on the right temporal area, 2.5 cm above the earlobe in the frontal plane passing through the acoustic meatus (position T8 of the 10–20 EEG positioning system^23^). This position allowed us to be facing the sylvian sulcus and the posterior insular cortex.^35,36^ The probe fixed on the graduate stick was inserted through a bone flap performed in the left temporoparietal area and through a small incision of the underlying dura contralateral to the right temporal stimulation. This experimental procedure allowed performing a transversal (left to right) trajectory for the probe through the 2 hemispheres and avoiding the resistance of the falx cerebri. The graduated stick was driven by the neurosurgeon through the head until its extremity reached a contact with the bone facing the center of the coil (Fig. 1C).

The first recording position Pc#1 was always the contact with the brain at the cranial entrance—ie, at the farthest position relative to the stimulus. Then, recordings were made by moving forward the probe from the left to the right side, by steps of 5 mm until it contacted the dura mater and the skull from the inside on the left side, where the coil was placed. During all the recording procedure, the graduated stick that served as a guide for the probe was maintained static by a mechanical device (Yasargil autostatic arm from B Braun Medical). We measured the distance corresponding to the first (entrance of the head) and the last (contact with the contralateral skull) positions of the probe. Because of tissue accumulation at the end of the experiments, the probe's extremity could not exactly reach a bone contact, and thus, the distance occupied by this residual parenchymal tissue was measured. Finally, we measured the bone and the scalp thickness under the coil.

### 2.5. Data processing and analysis

Raw data collected with the probe were in millivolts with the scale: 1V for 1.4 kilo-Tesla per second (kT/s). Raw data were processed using MATLAB software to correct the mean kT/s to T.

Statistical analysis was performed using GraphPad Prism (v.9). Outcomes are expressed as mean and 95% confidence intervals (CI), and appropriate effect sizes are systematically presented alongside *P*-values.

### 2.6. Feasibility to reach deep brain targets

Transcranial magnetic stimulation efficiency is related to the magnitude of the magnetic field to be delivered to the target. To treat patients with chronic pain, TMS intensity over the primary motor cortex is determined according to the patient's motor threshold (MT—ie, minimum intensity to induce a motor response by a single TMS stimulation). From a previous study conducted in our laboratory in 34 patients,^28^ we estimated the average minimal magnetic field capable of triggering a motor-evoked potential (MT) after stimulation of the healthy hemisphere. This average MT was 54% and was used here as the “reference” MT to reach a motor-evoked potential with the flat coil and to determine the required intensity to obtain the same MF for brain targets at different depth.

Using the participants' anatomical scans (3D T1-weighted MRIs—Siemens sequence; axial images acquired with voxel sizes between 0.938 and 1.0 mm), we determined the average depth of 6 clinically relevant brain targets,^13^ namely (1) the hand location in the primary motor cortex (M1; the center of the “omega-shaped” convolution within the central sulcus); (2) the posteroexternal part of the parietal operculum (S2); (3) the posterosuperior insula (PI, dorsal area of the anterior long gyrus, see Ref. 9); (4) the anterioventral part of insular sulcus (AI; insula pole); (5) the anterior midcingulate cortex (aMCC, rostral and pregenual area of the cingulate cortex, at a midpoint between the vertical line through the anterior commissure (VCA) and the genu of corpus callosum^38^); and (6) the dorsolateral prefrontal cortex (DLPFC, middle frontal gyrus corresponding to the lateral part of Brodmann areas 9 and 46, see Ref. 27).

Distance to the target (DTT) was defined as the minimal distance between the outer surface of the cortical target and the surface of the scalp.

We used MRIcron software (www.mricro.com), which provides a 3D surface rendering of the brain, to determine the individual coordinates (X, Y, Z) of the brain regions and the orthogonal coordinates of the scalp. Location of brain regions was defined based on an atlas of the human insula^9^ and the Human Connectome Project brain atlas.^15^ We used MATLAB scripts previously developed by our group to move from standardized space (MNI or Talairach) to individual space and define brain targets directly on the individual patient's anatomy (see Ref. 27 for more details).

The scalp-cortex distance was computed using the following formula: racine((Xscalp × voxel size − Xcortex × voxel size)^2 + (Yscalp × voxel size − Ycortex × voxel size)^2 + (Zscalp × voxel size − Zcortex × voxel size)^2). Scalp–cortex distances were averaged across subjects (±SD).

## 3. Results

### 3.1. Transmission on air

**Figure 2.**
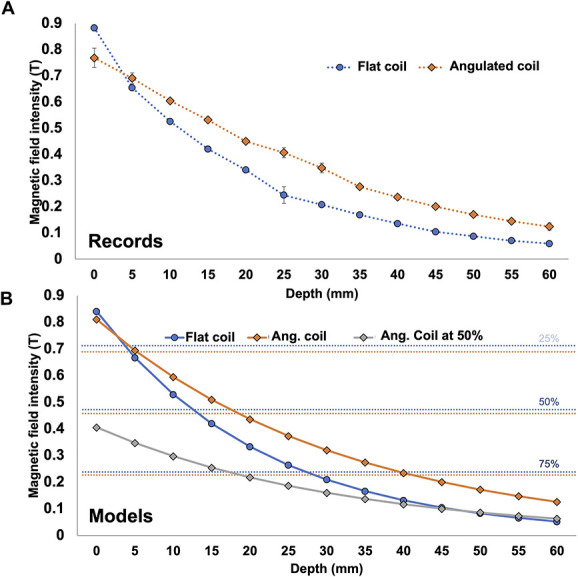
Magnetic field recordings of data in the air: (A) Average records with error bars (SD) for the flat coil (blue) and the angulated coil (orange) for a stimulation at 100% of power. (B) Fitting model corresponding to the flat coil (blue) and angulated coil (orange) for stimulations at 100% of maximal power, plus that obtained at 50% of maximal power (gray curve). The blue and orange horizontal lines represent the 20%, 50%, and 75% decrease thresholds when using the flat and angulated coils, respectively.

For the flat Cool-B65 coil, the maximum magnetic field was 0.88 T (95% CI: [0.86–0.90]), when the probe contacted the center of the coil. At 140 mm from the coil (farthest position), the field was measured at 0.0057 T (95% CI: [0.0056–0.0059]; Fig. 2A). The decay of the magnetic field with distance could be adequately fitted by a negative exponential curve with the following equation: F(x) = e^(−0.048.x)^. 0.8650229 (x = depth). The model showed a decrease of magnetic field of >50% at 1.5 cm and >75% at 3 cm (Fig. 2B).

For the angulated coil (Cool-DB80), the maximum magnetic field was 0.77 T (95% CI: [0.73–0.81]) at the first position, when the probe contacted the center of the coil. At 140 mm from the coils, the field was measured at 0.00993T (95% CI: [0.0111–0.00873]; Fig. 2A). The magnetic field could be best fitted by a reverse logarithmic curve presenting the following equation: F(x) = e^(−0.031.x)^. 0.810584 (x = depth). With this model, a decrease of magnetic strength of >50% was obtained at 2 cm and >75% at 4.5 cm (Fig. 2B).

### 3.2. Postmortem model

Cadaver specimens were 7 females and 3 males, at 7.1 days postmortem on average (range 1–12 days). The thickness of the scalp and the bone was 4.78 mm (95% CI: [3.87–5.69]) and 7.67 mm (95% CI: [6.58–8.75]), respectively. The first position of the probe at the entrance of the probe in the head was at 135.7 mm from the coil on average (95% CI: [125.41–145.98]), and its last position, closest to the coil at the contralateral stop, was 17.45 mm (95% CI: [13.38–15.42]).

**Figure 3.**
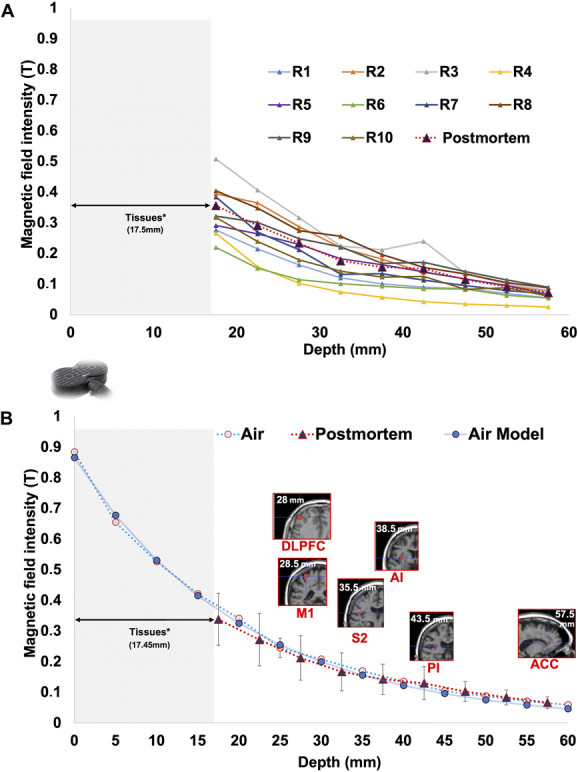
Magnetic field recordings in postmortem conditions measured in Tesla (T). (A) The 10 individual records with the mean decrease of the MF in red. (B) Here, the representation of the 2 mean curves of MF decay (error bars denote SD). The air model curve (blue) with the equation F(x) = e^(−0.048.x)^ as a function of the distance. Also represented the different cortical targets on an anatomical MRI according to their mean depth in mm. (*) For both (A and B) figures, in the postmortem condition, the nearest position to the magnetic coil was not zero, but 17.5 mm on average because of tissue accumulation in front of probe and interposition of meninges skull and scalp.

At the nearest intracerebral position, the (maximum) magnetic field was 0.34 T (95% CI: [0.285–0.39]). At the more distant position (entry in the skull contralateral to the coil), the magnetic field was measured at 0.0052 T (95% CI: [0.00441–0.00599], Fig. 3A). The magnetic field could be adequately fitted by a negative exponential function: F(x) = e^(−0.0418.x)^. 0.6156972 (x = depth). This model predicted a decrease of magnetic field of >50% at 1.5 cm and >75% at 3 cm (Fig. 3B).

### 3.3. Prediction of magnetic field strength at cortical targets

In the air and cadaver models, the fitting models were not significantly different (*P* = 0.80; Fig. 3B). Thus, for simplification of the estimation, but also because of large anatomic variability between the targets, we used the equation of air transmission to compute the intensity of MF at targets.

The average depth of the 6 possible cortical targets (see Methods, Table 1 and Fig. 3B) ranged from a minimal distance of 28 ± 1.41 mm for the DLPFC to a maximum of 57.5 ± 0.71 mm for the aMCC. No significant difference was observed between males and females.

**Table 1:** 1st column: depth of each cortical targets in mm; 2nd column: for each target, estimation of the magnetic field intensity (Tesla) produced by the classic flat coil after a stimulation over M1 target at the motor threshold (MT).

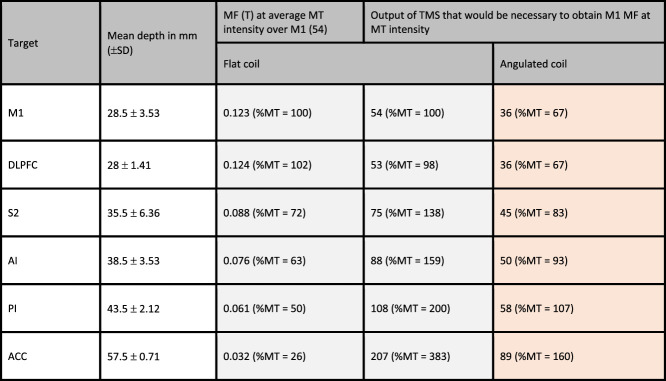

In brackets, the same results expressed as a % of the MT reaching the target. Third and fourth column: Each value refers to the output of the rTMS generator that would be necessary to induce an MF in the target equivalent to the MF measured in M1 after stimulation at the MT. In brackets, the same results expressed as a % of MT. Results are those measured in the postmortem brain for the classic flat coil (gray) and those extrapolated from the aerial conductions for the angulated coil (orange). All the measures presented here refer to an averaged output of rTMS generator set at the MT (54%) for a stimulation over M1 with the flat coil (see Methods).

ACC, anterior cingulate cortex; AI, anterior insula; DLPFC, dorsolateral prefrontal cortex; M1, primary motor cortex; PI, posterior insula; S2, secondary somatosensory cortex.

For the flat coil, the abacus in Table 1 provides the theoretical estimate of the magnetic field intensity delivered to each of the 6 targets if we stimulate at the reference MT (54%). Under such conditions, at M1, the magnetic field was estimated to be 0.12 T, whereas at the aMCC, the magnetic field would be of 0.032 T (ie, only 26% of the magnetic field delivered at M1). As shown in Table 1, the theoretical intensity of the rTMS generator needed to deliver a magnetic field equivalent to that of the motor cortex (=0.12 T) would rise from 54% of the rTMS generator power for M1 to 207% of maximal power of the rTMS generator for the aMCC. As another example, delivering a similar magnetic field to the posterior insula would require 108% of maximal power of the rTMS generator.

**Figure 4.**
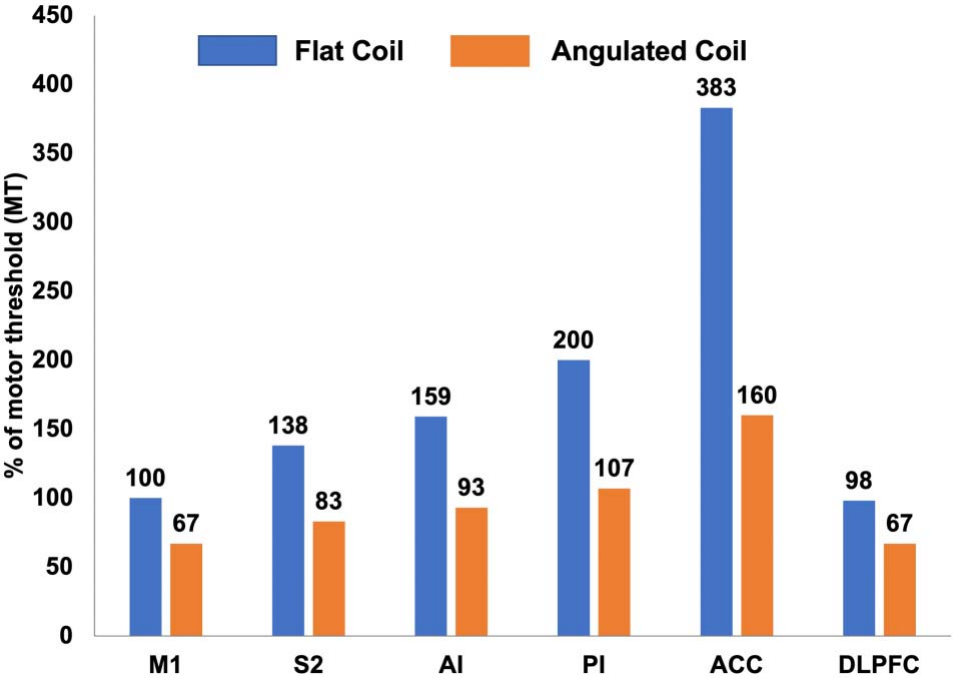
TMS intensities needed to be delivered at different cortical targets to obtain a magnetic field at a level equivalent to that used for motor threshold over M1. Blue and orange bars correspond to the flat and angulated coils, respectively. Values in percentage of maximum possible intensity of the device. ACC = anterior cingulate cortex; AI, anterior insula; DLPFC, dorsolateral prefrontal cortex; M1, primary motor cortex; PI, posterior insula; S2, secondary somatosensory cortex; TMS, transcranial magnetic stimulation.

For the angulated coil D-B80, a similar abacus was constructed (Table 1). The generator output necessary to reach a magnetic field equivalent in intensity to those present in M1 at different targets is shown in Figure 4.

For the flat coil, beyond >40 mm of depth (eg, for posterior insula and anterior cingulate cortices), the model finds a range of intensities impossible to be delivered by the rTMS stimulator (108% and 207%, respectively), whereas the angulated coil allows to stimulate the furthest target (Antérior Cingulate Cortex [ACC] = 57.1 mm) using reachable intensities with the rTMS stimulator (89%).

## 4. Discussion

This study provides direct recordings of the magnetic field produced by transcranial magnetic stimulation in human ex vivo head models and allows for a direct comparison of MF transmission with that in the air. The finding of a similar decay (Fig. 3B) in the 2 environments was expected considering the magnetic properties within biological tissues.^32^ This work is useful to estimate the magnetic field intensity (or “dose”) delivered on a given brain target.

Based on the average motor threshold determined in 34 patients who received rTMS in our laboratory, the average distance between brain targets and their nearest scalp point, and the calculated equation decays, we could estimate the intensity needed to reach several intracranial targets, relative to intensity needed to obtain the motor threshold after stimulation of M1. Our results showed a rapid and important decrease of MF intensity with distance, with a > −30% drop at distances consistent with the S2 area (35 mm), −50% for the posterior insula (∼43.5 mm), and up to −75% for the aMCC (∼57 mm), when using a flat coil. Given such important decrease, it is highly unlikely that rTMS delivered at MT would be able to effectively stimulate these targets. A simple option to counterbalance this decay of magnetic field would be to increase the power of stimulation, but this alternative presents with both practical and clinical difficulties. As shown in Figure 4, the required adaptation of intensity is important and would require stimulation intensities far above the MT for a number of relatively deep targets such as the insula or ACC (Table 1), with the risk of triggering epileptic seizures.^19^ Our results also suggest that an angulated coil may be more appropriate for reaching deep targets without requiring excessive intensity increase (Table 1) and would represent a good alternative to a flat coil when stimulating targets located more than 40 mm from the surface. This is a finding in agreement with previous work based on mathematical modeling of electric fields induced by magnetic flux per surface unit (E = DΦ/Dt, Φ being the magnetic flux), which also reported the superiority of the angulated coil, compared with flat coils, in reaching deep targets (Deng et al.^7^). Deng et al.^6^ predicted a linear decay of MF when “the coil was large relative to the head,” which was the case in their modeling approach, but clearly not when using a real coil over real heads, as was the case in our experiments, which found a negative exponential decay.

Because rTMS of M1 is effective in around 50% of cases with intractable pain,^12^ targeting other cortical areas distant from the surface of the skull may become a new challenge for pain research. The 6 potential targets tested here have shown brain activity abnormalities in neuropathic pain,^10,11,26^ and thus, can be considered as potential targets for rTMS to induce pain relief. This study suggests that most of these alternative targets may be difficult to reach except by using stimulus intensities far above the MT. This option is however challenging because of higher incidence of unpleasant contractions of cranial muscles, spurious electrical currents in brain surfaces, and risk of inducing epileptic seizures. The preferential use of an angulated coil for deep structures seems as a safer and feasible alternative. A possible and rapidly evolving third strategy to safely reach deep targets may be based on indirect stimulation through the activation of surface structures known to be anatomically connected with deeper structures.^16,34^ Although highly attractive, this option depends on both white matter tracts and their demonstration with neurophysiological techniques, which remains largely incomplete for many of the deep potentially relevant structures.

## 5. Limitations

The estimations presented here seemed as an appealing alternative to the ideal situation of field recording in living subjects, which are not possible to date, but have a number of limitations. The model of postmortem brain is necessarily different from living tissues, and the recording position in this study is different from that in clinical rTMS sessions with patients. Measures in cadavers were made in decubitus, as is performed in surgery under stereotactic approach, whereas TMS is performed in general in sitting position. Also, although craniectomy certainly induced a CSF leakage, CSF may not significantly impact the magnetic decay, which depends mainly on the distance from the source and not on the quality of the intermediate tissues.^31^ Even we could not record the secondary electric currents induced by TMS pulses, the composition of the living tissues and their electrical properties is different, and the electrode induction differs from the postmortem model.

The use of M1 motor threshold as the reference intensity to induce neurophysiological effects is another limitation because it cannot be extrapolated to other regions such as the prefrontal or the operculoinsular cortices. In the case of the insula, the different cytoarchitectonic structure between its anterior and posterior parts^24^ could make the properties of electric induction and neuromodulation different. These differences in inter-regional excitability need to be considered for development of new rTMS strategies because testing other paradigms than those previously validated may expose to epileptic seizures. For instance, a combination of 50-Hz and 5-Hz pulses (“theta burst” stimulation) focusing the insula was recently reported to induce seizures in 2 healthy subjects.^20^ Increasing MF strength to reach deep targets may entail similar risks of superficial cortical layers under the coil.

## 6. Conclusions

This study provides an experimental quantification of the magnetic field decay according to the distance from the skull, based on magnetic recordings on postmortem models. It confirms a nonlinear decay of the magnetic field, which is not affected by biological tissues, relative to the air. Our work allows to estimate the MF intensity to be delivered to reach different targets within the brain and points out the difficulties to reach deep targets such as the insular or cingulate cortices with flat rTMS coils. As previously suggested, the angulated coil seems superior to the flat coil to reach deep targets (>2.5 cm of depth). Our work emphasizes the need to adapt the magnetic stimulation to the depth of the targets, the potential advantages of angulated coils, and provides an abacus relating the field decay and intracranial target depth for rTMS users.

## Disclosures

The authors have no conflict of interest to declare.
